# A successful antenatal outcome in a patient with refractory Takayasu’s arteritis treated with tocilizumab throughout pregnancy—a case report

**DOI:** 10.1093/omcr/omad046

**Published:** 2023-05-30

**Authors:** Behram Khan, Trixy David, Jon Sussman, Louise Simcox, Clare Tower, Calvin Soh, Ian N Bruce

**Affiliations:** The Kellgren Centre for Rheumatology, Manchester Royal Infirmary, Manchester University NHS Foundation Trust, Manchester, UK; The Kellgren Centre for Rheumatology, Manchester Royal Infirmary, Manchester University NHS Foundation Trust, Manchester, UK; NIHR Manchester Biomedical Research Centre, The University of Manchester, Manchester Academic Health Science Centre, Manchester, UK; Department of Neurology, Greater Manchester Neuroscience Centre, Salford, Greater Manchester, UK; Saint Mary’s Hospital, Manchester University NHS Foundation Trust, Manchester, UK; Maternal and Foetal Health Research Centre, The University of Manchester, Manchester Academic Health Science Centre, Manchester, UK; Saint Mary’s Hospital, Manchester University NHS Foundation Trust, Manchester, UK; Maternal and Foetal Health Research Centre, The University of Manchester, Manchester Academic Health Science Centre, Manchester, UK; Department of Radiology, Manchester University NHS Foundation Trust, Manchester, UK; The Kellgren Centre for Rheumatology, Manchester Royal Infirmary, Manchester University NHS Foundation Trust, Manchester, UK; NIHR Manchester Biomedical Research Centre, The University of Manchester, Manchester Academic Health Science Centre, Manchester, UK

## Abstract

Takayasu’s arteritis (TA) is a rare form of large-vessel vasculitis for which tocilizumab (TCZ) may be administered in resistant or refractory disease. Current British Society of Rheumatology advice is to stop TCZ 3-months pre-conception. We report the case of a 33-year-old woman with extensive TA treated with TCZ, azathioprine and glucocorticoids in pregnancy. She was closely monitored with MDT input and TCZ was continued throughout pregnancy as the benefits were thought to outweigh the risks. Our case also highlights the importance of accurate blood pressure monitoring in an appropriate anatomical location, given the extent of her disease. Our patient’s disease remained stable throughout the antenatal and post-partum period with a successful pregnancy outcome and no maternal or foetal complications. TCZ is suitable for select cases of refractory TA during pregnancy.

## INTRODUCTION

Takayasu’s arteritis (TA) is a large vessel vasculitis of unknown aetiology. Glucocorticoid therapy is a mainstay of treatment. More recently, tocilizumab (TCZ), a human monoclonal antibody that competitively inhibits the binding of IL-6 to its receptor, has been approved in resistant or refractory disease [1]. Because of the limited data in pregnancy, current British Society of Rheumatology guidance is to stop TCZ 3 months prior to conception [2].

### CASE REPORT

We report a case of a 33-year-old Caucasian lady (Gravida 2, Para 1) with TA treated with TCZ throughout pregnancy at a tertiary specialist Foetal and Maternal Medicine—Rheumatology combined clinic.

**Figure 1:**
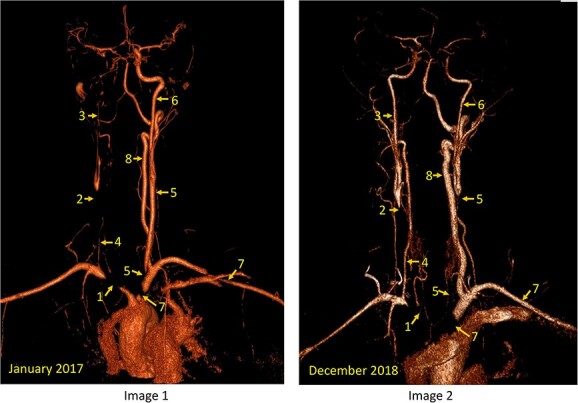
Comparison of the MR angiographic findings between January 2017 (Image 1) and December 2018 (Image 2). The arteries are labelled numerically as follows: brachiocephalic artery (1), initially stenosed, subsequently progressed with occlusion from aortic arch to the vertebral artery origin. Right common carotid artery (2) was severely narrowed with only a trickle flow proximally and occluded up to the carotid bifurcation, whereas the right internal carotid artery (3) is severely narrowed initially and shows reconstitution on subsequent scan. Right vertebral artery (4) was initially severely attenuated, with only a trickle flow, and shows reconstitution on subsequent scan and appeared almost normal. Left common carotid artery (5) was initially severely narrowed over a short segment from the aortic arch origin becomes occluded from the arch to the carotid bifurcation, whereas the left internal carotid artery (6), initially normal, subsequently shows moderately severe narrowing in the neck. The left subclavian artery has a short segment of severe stenosis, which had progressed slightly, whereas the distal subclavian artery narrowing in the supraclavicular fossa had resolved. Left vertebral artery (8) is dominant and was not affected initially, and becomes hypertrophic on subsequent scan.

The patient gave a 4-year history of daily headache with photophobia diagnosed previously as migraine. In January 2017, she presented with 3 days of malaise with typical headache and right sided upper limb aching with incoordination. The following day she presented with repeated 10-min episodes of left facial and upper limb weakness and word-finding difficulties. She was reviewed by neurology team and her workup suggested a right carotid territory dissection with parietal cortex involvement on MR imaging; however, MR angiography revealed widespread large vessel vasculitis with proximal right brachiocephalic, right common carotid artery, left carotid artery origin and left Subclavian involvement (Fig. 1, Image 1). Given the findings she was diagnosed with TA.

Initial treatment was with intravenous (IV) methylprednisolone 1 gram and IV cyclophosphamide. IV methylprednisolone was given for 5 days followed by 60 mg oral prednisolone. The inflammatory markers improved but had not normalized. Following the seventh course of cyclophosphamide, she had a left middle cerebral artery embolic event in late 2017. Angiogram showed progression of the disease in the major arteries of the neck (Fig. 1, Image 2). Because of refractory disease, her treatment was changed to subcutaneous TCZ (162 mg weekly), azathioprine and a weaning dose of prednisolone (25 mg). Azathioprine was started at 50 mg daily and escalated to 100 mg. She conceived on this combination and was assessed first time in our combined Rheumatology/Obstetrics clinic at 14 weeks gestation (March 2019). She had no other co-morbidities and was a non-smoker. Baseline blood pressure (BP) in her thigh was 130/84 mmHg and 90/68 and 67/53 mmHg in her left and right arms, respectively. Her Indian Takayasu Clinical Activity Score (ITAS) at first pregnancy visit was 0 (scored retrospectively by INB) [3] and her CRP was 5 mg/l (normal < 5.0). Given her extensive disease and previous life-threatening complications and following detailed discussion with the patient and her partner, a decision to continue TCZ was made, with close monitoring and multi-disciplinary support. A steroid-taper of 5 mg every 4 weeks was initiated, and she was started on aspirin 150 mg at night and tinzaparin 4500 IU daily.

BP monitoring used her thigh or calf and at 22 weeks, amlodipine 5 mg daily was added as her BP was 170/70 mmHg. Ultrasound assessments of placental function were performed at 17 and 22 weeks. There was normal foetal growth and both uterine artery dopplers demonstrated normal resistance waveforms. Because of risk of pre-eclampsia urine protein ratio was check at each clinic visit and this remained within normal range (0–30 mg/mmol).

Prednisolone was further tapered to 7.5 mg daily by 34 weeks and maintained through the rest of her pregnancy. Systemically, she had no TA flares; at her 34-week visit her ITAS score remained 0 and her CRP was <1.0 mg/L. There were concerns over the risks of vaginal delivery, and an elective caesarean section (CS) was planned at 39 weeks gestation. However, spontaneous labour occurred 2 days before her scheduled admission date and she had an emergency CS. She delivered a baby girl weighing 2768 grams with no peri-partum maternal or foetal complications. TCZ and azathioprine were withheld during the peri-partum period for 2–3 weeks until her CS wound healed and she had IV hydrocortisone 25 mg three times daily for 48 h and then prednisolone was continued at 7.5 mg daily.

Few months later (2020), she underwent MRA brain and carotid to assess disease status. Imaging was discussed at neuroradiology multidisciplinary team meeting; the stenotic lesion remained stable and there were no radiographic features to suggest disease progression.

Subsequently she has been maintained on TCZ and AZA and her prednisolone was tapered to 5 and 7.5 mg alternate days. More recent imaging (2022) confirms disease stability. At the time of reporting, her baby is 40 months old and is healthy.

## DISCUSSION

Relapsing, resistant TA poses significant challenges during pregnancy. Whilst biologics are discontinued prior to pregnancy or early in the first trimester; uncontrolled or flaring disease can put both the mother and foetus at risk of complications such as worsening hypertension, pre-eclampsia, prematurity and/or intra-uterine growth restriction [4]. High-dose glucocorticoids also contribute to BP dysregulation and impaired glucose tolerance, thus adding to overall pregnancy risks.

TCZ provides good disease control and is steroid sparing. No relevant placental transfer of IgG1 occurs in the first trimester; however, transfer can increasingly occur through the second and third trimesters [4]. Current guidelines suggest stopping TCZ 3 months prior to conception [2].

Data regarding TCZ safety in pregnancy are mainly from RA patients. One case series of prenatal or early first trimester exposure to TCZ found that in 180 prospective and 108 retrospective cases there was no substantial increase in the rate of congenital malformations [5]. The rate of spontaneous abortion (21%) was similar to the general population (10–20%) [6]. A retrospective analysis of 61 Japanese RA patients on TCZ found that the rate of spontaneous abortion (18%), congenital abnormalities (none) and other adverse pregnancy outcomes (10%) were similar to the general population [7]. In this study, only one patient continued TCZ throughout pregnancy and delivered a baby with a low birth weight (<2000 g).

We found two cases of patients taking TCZ in pregnancy for TA [4, 8]. As with our case, both had refractory disease with extensive damage. TCZ was continued throughout pregnancy in one patient and stopped at 6 weeks gestation in the other. Pregnancy outcomes were favourable apart from one case developing neonatal jaundice [8].

More recently TCZ has been widely used for the management of severe COVID-19 [9]. Ten pregnant patients were included in trial; however, no specific maternal and neonatal outcomes were noted. In another retrospective study of 12 pregnant women with COVID-19 receiving TCZ, all pregnancies resulted in live births, there were two reports of transient hepatotoxicity and one of cytomegalovirus [9]. To gain further understanding and effect of immunomodulators on pregnancy and neonatal outcome, a more robust regulatory structure for recruiting pregnant patient in clinical trial is warranted.

Our case had aggressive TA and since the benefits of continuing TCZ to maintain stable disease outweighed the theoretical risks, TCZ was continued through pregnancy with a successful outcome. This case also highlights the need for accurate monitoring BP in an appropriate anatomical location guided by angiographic findings. In selected cases, TCZ therefore has a role in managing refractory TA in pregnancy.

## CONCLUSION

In selected cases, TCZ has a role in managing refractory TA in pregnancy. It is pertinent to monitor BP accurately in an appropriate anatomical location guided by angiographic findings. Our case report also highlights the importance of multidisciplinary team role in managing such complex cases.

## FUNDING

No specific funding was received from any bodies in the public, commercial or not-for-profit sectors to carry out the work described in this manuscript.

## ETHICAL APPROVAL

No approval required.

## CONSENT FOR PUBLICATION

Written informed consent was obtained from the patient for publication of this case report and any accompanying images. A copy of the written consent is available for review by the editor of this journal.

## GUARANTOR

Dr Behram Khan.

## AUTHOR CONTRIBUTIONS

All authors contributed to writing, literature review and critical analysis of this case report. The final manuscript was approved by all authors.

## CONFLICT OF INTEREST STATEMENT

None declared.

## DATA AVAILABILITY

Data resulted from this study are available from the corresponding author on reasonable request.
